# Synthesis, photophysical and electrochemical characterization of terpyridine-functionalized dendritic oligothiophenes and their Ru(II) complexes

**DOI:** 10.3762/bjoc.9.100

**Published:** 2013-05-06

**Authors:** Amaresh Mishra, Elena Mena-Osteritz, Peter Bäuerle

**Affiliations:** 1 Institute of Organic Chemistry II and Advanced Materials, Ulm University, Albert-Einstein-Allee 11, 89081 Ulm, Germany

**Keywords:** dendrons, electrochemistry, oligothiophene, spectroscopy, terpyridine

## Abstract

Pd-catalyzed Sonogashira cross-coupling reactions were used to synthesize novel π-conjugated oligothienylene-ethynylene dendrons and their corresponding terpyridine-based ligands. Their complexation with Ru(II) led to interesting novel metallodendrimers with rich spectroscopic properties. All new compounds were fully characterized by ^1^H and ^13^C NMR, as well as MALDI–TOF mass spectra. Density functional theory (DFT) calculations performed on these complexes gave more insight into the molecular orbital distributions. Photophysical and electrochemical studies were carried out in order to elucidate structure–property relationships and the effect of the dendritic structure on the metal complexes. Photophysical studies of the complexes revealed broad absorption spectra covering from 250 to 600 nm and high molar extinction coefficients. The MLCT emission of these complexes were significantly red-shifted (up to 115 nm) compared to the parent [Ru(tpy)_2_]^2+^ complex.

## Introduction

Research concerning the design, characterization and application of organic semiconductors is carried out intensively due to the attractive prospects of their application in organic and molecular electronics [1–5]. In particular, thiophene-based oligomers and polymers are amongst the best-studied π-conjugated systems in the past few decades because of their tunable optoelectronic properties [6–8].

Furthermore, transition-metal complexes offer significant advantages such as long-lived luminescent excited states, high chemical and photochemical stabilities, and tunability of the excited-state energies [9]. Additionally, they can be employed as energy donor or acceptor units in electronic energy transfer processes [10]. In particular, ruthenium(II) polypyridine complexes have been extensively studied and represent an area of widespread interest that has found applications in opto-electronic devices and nanotechnology [11–13]. The stability of these transition-metal complexes is generally attributed to the σ-donor/π-acceptor character of the dative metal–nitrogen bond. These Ru(II) complexes possess various important photophysical features that arise from the population of a triplet luminescent level as the lowest excited-state after photoexcitation [14–16], and their derived multinuclear complexes are topologically interesting species including rods, wires, helicates, and dendrimer scaffolds [16–22]. In the past years, a large number of luminescent dendrimers based on polynuclear transition-metal complexes have been developed as promising materials for the study of unidirectional energy transfer and multielectron-transfer processes as well as for light-harvesting applications [19].

Among the polypyridine ligands, 2,2':6',2"-terpyridine (tpy) has been used extensively, despite the low luminescence displayed by these complexes, due to the interesting geometry of the tpy ligand, which allows axial type arrangement of the substituents placed on the 4'-position of the coordinated tpy. Among them, ligands of the tpy-ethynyl type have proven to be very useful and provide a library of photoresponsive complexes derived from the basic Ru(II)-tpy type module [23–27]. Recently, tpy-complexes that incorporate π-conjugated thiophene or thienylene-ethynylene units became available, and the study of their photophysical properties is of great relevance, given the importance of oligothiophene and tpy-units as electro- and photoactive conjugated materials [21,28–32]. Besides these linear counterparts, various tpy-containing polymeric and dendritic structures have been developed and their photophysical properties studied [33–38]. However, to our knowledge, tpy-functionalized dendritic oligothiophenes have not been synthesized so far. The complexation of these dendrons could lead to interesting nanosized materials containing different chromophores to study electronic energy-transfer processes.

A series of shape-persistent *all*-thiophene dendrons and dendrimers were recently introduced by Advincula et al. [39–40] and us [41–42]. These dendritic oligothiophenes are excellent model compounds for the study of fundamental photophysical and electronic properties. Furthermore, core-functionalization of oligothiophene dendrons with optically and/or redox active perylenebisimide, phthalocyanine and quinoxaline units have been developed as possible light-harvesting molecules [43–45]. We report herein the synthesis of oligothienylene-ethynylene based 3-dimensional molecular materials, namely metallodendritic structures, functionalized with tpy-chelating ligands and the corresponding Ru(II) complexes. Thorough photophysical and electrochemical investigations provide an insight into the electronic structure of the novel materials, which represent an interesting extension of recently introduced *all*-thiophene dendrons. The core functionalization with a redox and optically active ligand and a corresponding metal complex and the extension of the conjugated π-system via stiff triple bonds [42] should lead to complex 3D organic semiconductors with spatially defined functionalities.

## Results and Discussion

**Scheme 1:**
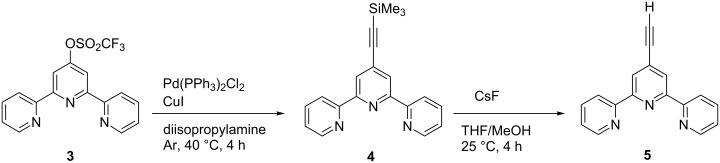
Synthesis of 4'-ethynyl-2,2':6',2''-terpyridine (**5**).

**Syntheses.** A modular approach was selected for the synthesis of the targeted metallodendrimers, consisting of (1) the preparation of dendrons by employing Sonogashira cross-coupling reactions, (2) their functionalization with tpy-ligands, and (3) formation of the corresponding homoleptic Ru(II) complexes. As a coupling component, tpy **5** was synthesized starting from the corresponding tpy-triflate **3**, which was obtained from 4'-hydroxy-2,2':6',2''-terpyridine in 74% yield according to a literature procedure [46]. Reaction of tpy **3** with trimethylsilylacetyelene (TMSA) afforded 4'-trimethylsilylethynyl-2,2':6',2''-terpyridine (**4**) [47], which was readily deprotected by CsF in methanol to give the targeted 4'-ethynyl-2,2':6',2''-terpyridine (**5**) (Scheme 1).

**Scheme 2:**
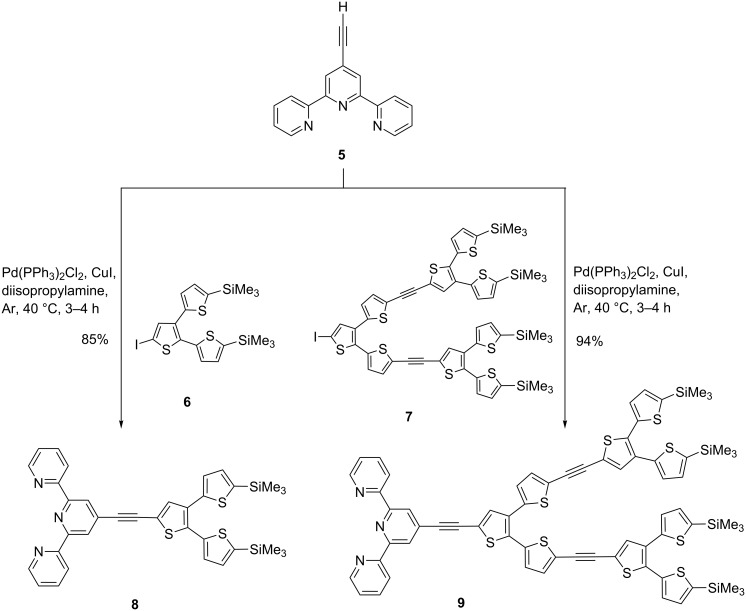
Synthesis of tpy-functionalized dendritic oligothiophenes **8** and **9**.

Cross-coupling of ethynylated tpy building block **5** with iodinated G1-dendron **6** and G2-dendron **7** [42] under standard Sonogashira-type coupling conditions afforded key tpy-functionalized dendritic oligothiophenes **8** and **9** in 85 and 94% yield, respectively (Scheme 2).

**Scheme 3:**
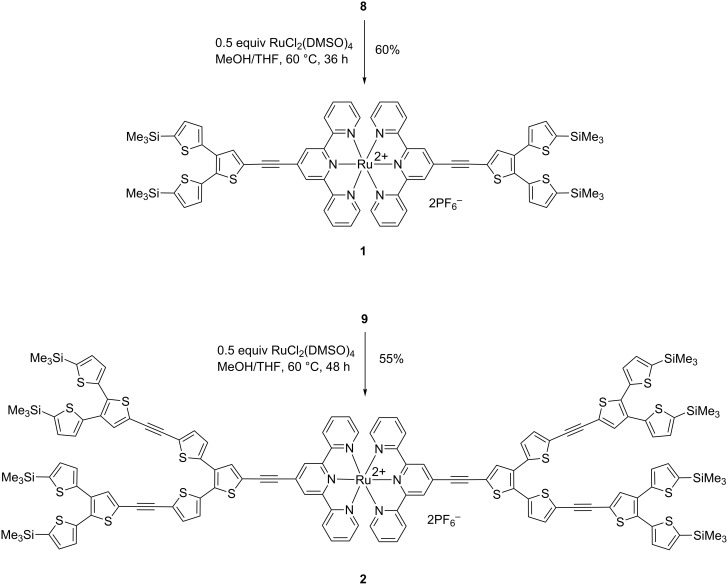
Synthesis of homoleptic Ru(II) complexes **1** and **2**.

In a final step, tpy-ligands **8** and **9**, which are soluble in common organic solvents, were then reacted with half an equivalent of [RuCl_2_(DMSO)_4_] in a mixture of MeOH/THF to afford the corresponding homoleptic Ru(II) complexes **1** and **2** in 55–60% yield as red solids after chromatographic purification. Anion exchange to form the corresponding hexafluorophosphates was achieved through precipitation from a saturated methanolic solution of NH_4_PF_6_ (Scheme 3). All compounds were characterized by NMR, MALDI–TOF-mass spectrometry and elemental analysis as well as by cyclic voltammetry and UV–vis spectroscopy.

**Electronic absorption and emission properties.** One of our goals was to study electronic communication between the core metal complex and the attached oligothiophene dendron as a function of increasing generation. Therefore, UV–vis absorption and luminescence spectra of ligands **8**, **9** and metallodendrimers **1**, **2** were measured and analyzed (Table 1).

**Table 1:** Absorption, fluorescence and quantum yield data for the ligands and complexes in acetonitrile solution.

	λ_abs_ (nm) (ε/10^−4^ L mol^−1^ cm^−1^)	λ_em_ (nm)	Φ_f_

**8**	285 (4.4), 314 (3.01), 364 (2.59)	465	0.15
**9**	278 (5.73), 325 (5.09), 371 (5.72)	541	0.24
**1**	275 (8.43), 314 (8.40), 331 (7.85), 370 (4.22), 509 (8.04)	693	~7 × 10^−4^
**2**	276 (12.05), 315 (12.23), 335 (11.90), 369 (11.68), 513 (8.66)	755	~1 × 10^−4^
[Ru(tpy-th)_2_][PF_6_]_2_^a^	283 (5.30), 316 (6.00), 332 (5.50), 499 (2.60)	670	1 × 10^−4^
[Ru(tpy)_2_][PF_6_]_2_^a^	270 (3.20), 307 (5.20), 474 (1.04)	629	<5 × 10^−6^

^a^Data taken from [48]; (th = thiophene).

**Figure 1:**
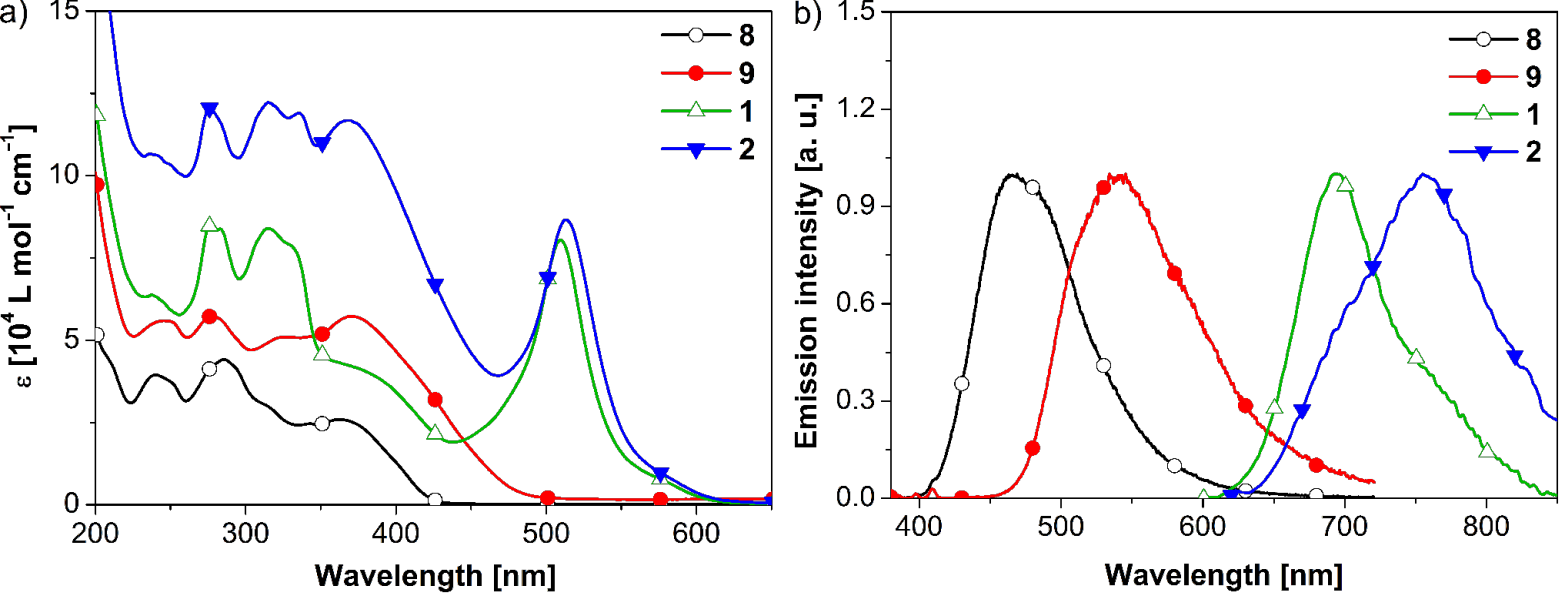
(a) Absorption spectra of ligands and their corresponding Ru(II)-dendrimers in acetonitrile solution at 293 K. (b) Normalized emission spectra of ligands (excited at λ_exc_ = 365 nm), and the corresponding Ru(II)-complexes (excited at λ_exc_ = 510 nm), in dilute deaerated acetonitrile solution at 293 K.

Absorption spectra of ligands **8**, **9** and complexes **1**, **2** in air-equilibrated acetonitrile at 293 K are shown in Figure 1a and summarized in Table 1 together with reported data for the parent [Ru(tpy)_2_]^2+^ complex [48]. Ligands **8** and **9** showed absorption maxima between 260 and 300 nm that correspond to ligand-centered (LC) π→π^*^ transitions, whereas the absorptions at 320 to 400 nm belong to the π→π^*^ transitions of the tpy-functionalized thienylene-ethynylene moieties [26]. In comparison with the absorption spectra of dendrons **6** and **7** [42], a significant spectral broadening was observed for these ligands, indicative of extended conjugation and more delocalized electronic distribution between tpy and ethynylene-thienylene chromophores. The results were further supported by theoretical calculations (see below). For ligands **8** and **9**, with increasing generation of the dendron from G1 and G2, a slight red-shift in the lowest energy absorption maximum and a significant enhancement in the molar absorption coefficient were observed. Furthermore, the absorption band of ligand **9** containing G2-dendron was significantly broadened due to the presence of multiple chromophoric units [42].

Absorption spectra of complexes **1** and **2** showed features that are readily attributable to the spin-allowed ligand-centered (LC) π→π* bands in the UV region and the singlet metal-to-ligand charge-transfer (^1^MLCT) bands corresponding to the parent [Ru(tpy)_2_]^2+^ complexes (up to 300 nm and ~500 nm) (Figure 1a and Table 1) [16,48]. For both metallodendrimers, no significant shifts were observed for the ligand-centered absorption bands, while the molar extinction coefficient increased due to the increased 1:2 ligand stoichiometry. The intense bands in the 420–550 nm region are due to ^1^MLCT (dπ→π*) transitions centered on the ruthenium moiety [16,49]. The position of the ^1^MLCT absorption maximum was at 509 nm for **1** and 513 nm for **2** concomitant with a large increase in molar absorptivity for both complexes (ε ~ 8 × 10^4^ L mol^−1^ cm^−1^) compared to the parent [Ru(tpy)_2_]^2+^ (λ_abs_ = 474 nm, ε ~ 1.04 × 10^4^ L mol^−1^ cm^−1^). These results can be explained in terms of an extended and efficient delocalization of the charge at the coordinated ligands. Independently, Beley et al. and Constable et al. reported that the attachment of the 2-thienyl group in the [Ru(tpy-th)_2_]^2+^ complex (th = thiophene) causes a red-shift in the absorption maximum (λ_abs_ = 499 nm, ε ~ 2.6 × 10^4^ L mol^−1^ cm^−1^) and only a minor change in the molar absorption coefficient with respect to the parent [Ru(tpy)_2_]^2+^ [48,50]. As a result, these dendritic oligothienylene ethynylene-functionalized Ru(II) complexes can be considered as better light-harvesting groups than the parent Ru(II) complex because of their enhanced absorption in the whole UV–vis region.

Ligands **8** and **9** showed single and broad emission maxima centered at 465 and 541 nm, respectively. As shown in Figure 1b the band maximum undergoes a marked bathochromic shift with increasing size and also compared to the parent dendrons **6** and **7**, which further ascribed to the extended conjugation. The fluorescence quantum yields (Φ_f_) of the ligands were found to be 0.15 and 0.24, respectively. Excitation of complexes **1** and **2** at their respective ^1^MLCT band in deaerated acetonitrile resulted in weak emissions at 693 nm and 755 nm and extended to the near-infrared region. For complexes on the basis of their structureless emission and low fluorescence quantum yields (Φ_f_ ~ 10^−3^ to 10^−4^), the bands were attributed to the radiative deactivation of the lowest ^3^MLCT excited state of the Ru-based moieties [18,25,50]. It is interesting to note that for both metallodendrimers, **1** and **2**, the emission bands are significantly red-shifted compared to the parent [Ru(tpy)_2_]^2+^ complex (Δλ_em_ = 64 and 126 nm, respectively, Table 1) [48], indicating that the ^3^MLCT luminescence energy levels in these complexes undergo a stabilization with increasing size of the thienylene-ethynylene units [18]. Beley et al. reported a similar red-shift of the ^3^MLCT emission band for the [Ru(tpy-th)_2_]^2+^ complex (Δλ_em_ = 41 nm) compared to the parent [Ru(tpy)_2_]^2+^ (see Table 1) [48]. Recently, Andvincula et al. also reported a large red-shift (Δλ_em_ = 165 nm) of the ^3^MLCT emission for the ruthenium(II)-cored phenanthroline-oligothiophene dendrimer compared to the parent phenanthroline complex [51]. This unusually large red-shift indicates that the thiophene dendron has very strong electronic interactions with Ru-to-ligand CT states and strongly stabilizes these MLCT states.

Additionally, the luminescence spectra of both complexes **1** and **2** were measured by exciting at a higher energy band at ~370 nm, which corresponds to the π–π* transition of the ligand. Interestingly, the results show effective quenching of the ligand-based emission and bands centered at the characteristic emission maxima of complexes **1** and **2**, indicating efficient photoinduced energy transfer from the oligothiophene to the nonemissive [Ru(tpy)_2_]^2+^ center of the metallodendrimer.

**Figure 2:**
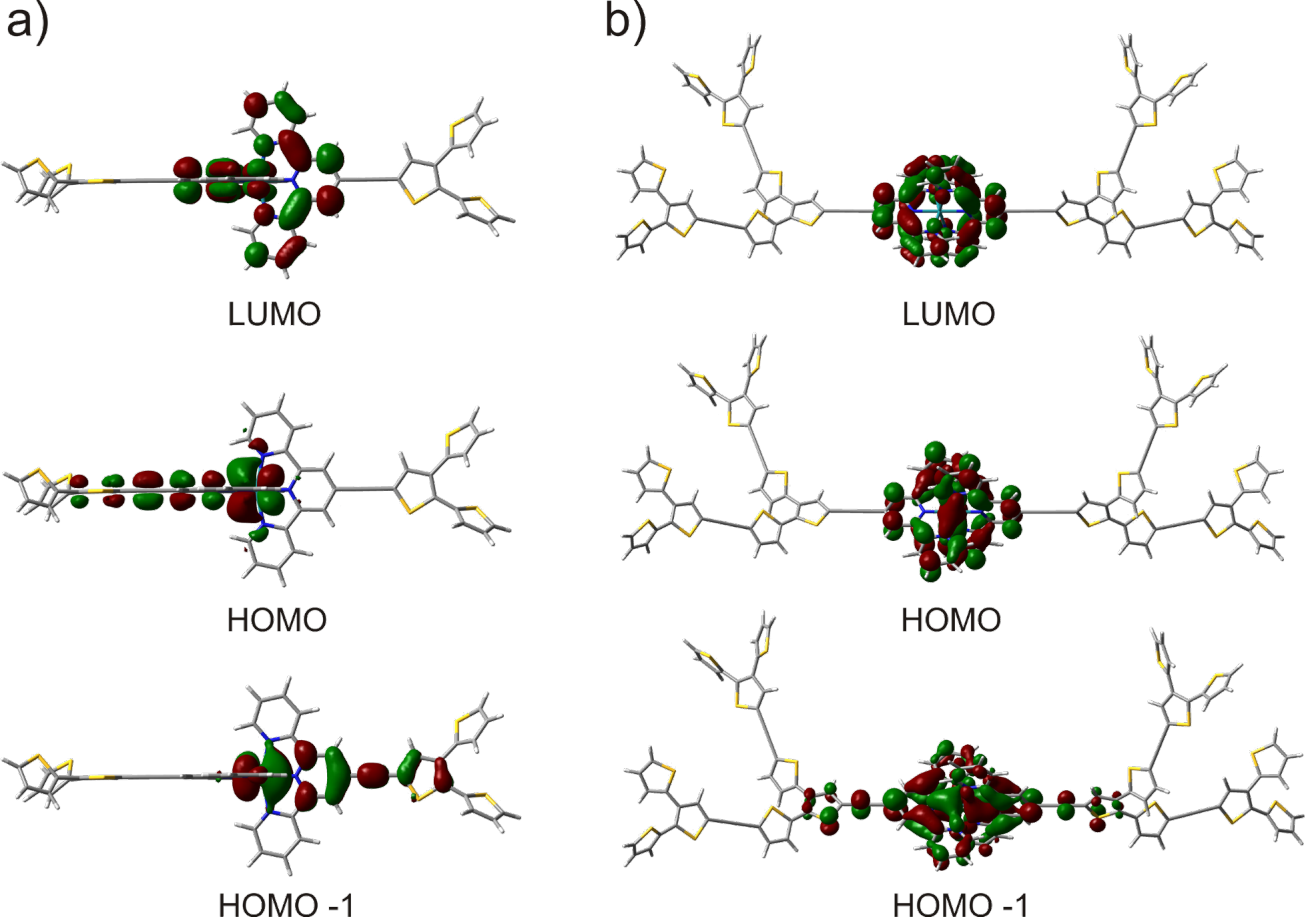
Electronic distribution of the frontier orbitals (HOMO, HOMO-1 and LUMO) for complexes **1** (a) and **2** (b).

**Quantum chemical calculations.** In order to gain insight into the structural properties, the electron density distribution of the frontier orbitals of both complexes was analyzed by density functional theory (DFT) using the B3LYP/LANL2DZ basis set (Figure 2). For both complexes the tpy-acetylene-thiophene units are coplanar thereby providing an extended conjugation pathway from the tpy ligand to the oligothiophene dendron. Due to the high molecular symmetry, the highest occupied molecular orbitals (HOMO and HOMO-1) for **1** were found to be degenerate. These orbitals are delocalized from the Ru-center to the adjacent ethynylene-thiophene units. In contrast, the first occupied molecular orbital (HOMO) in complex **2** is exclusively delocalized on the pyridine and Ru-metal center. The HOMO-1 is rather delocalized over the Ru-center to the ethynylene-thiophene unit. In turn, the low lying HOMO-3 and HOMO-4 (not shown in Figure 2) are distributed over the oligothiophene dendrons. In both complexes the LUMO has exclusive contributions from both tpy-ligands. From the molecular orbital analysis, the lowest energy transition between the HOMO (and degenerated HOMO-1 in the case of **1**) and the LUMO for both complexes corroborate the ^1^MLCT character.

**Figure 3:**
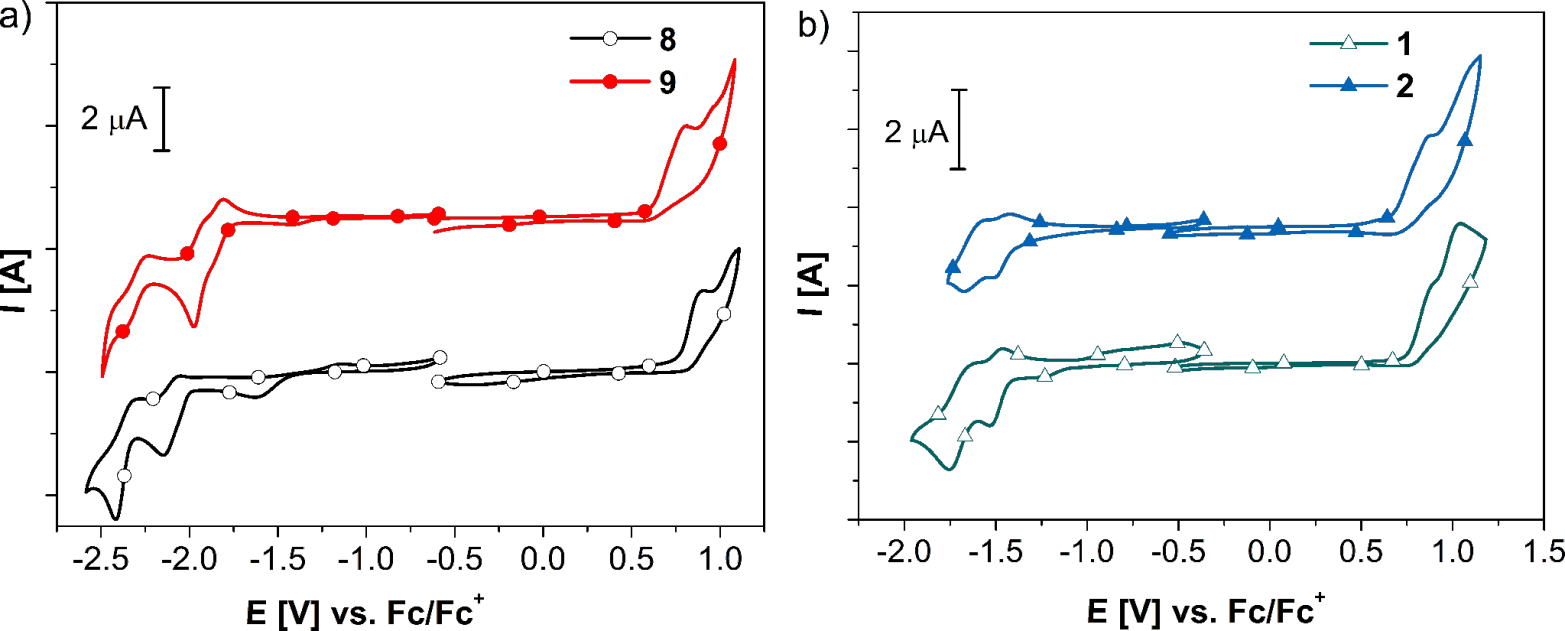
CV of 1.0 mM solutions of ligands (a) and complexes (b) in anhydrous dichloromethane (oxidation) and anhydrous DMF (reduction) by using TBAPF_6_ (0.1 M) as a supporting electrolyte at 298 K under an argon atmosphere. Potentials are given against an internal Fc/Fc^+^ reference. The scan rate was 100 mV/s.

**Redox properties.** Oxidation and reduction properties of all compounds were investigated by using cyclic voltammetry (CV). Electrochemical data of the ligands and the corresponding Ru(II) complexes are presented in Table 2. The attachment of tpy*-*ligands to the dendrons resulted in the appearance of multiple quasi-reversible one-electron reduction processes. The CV response of ligand **8** in the negative potential regime showed the typical two quasi-reversible waves due to successive one-electron reductions of the tpy unit [14,34–35]. In contrast, ligand **9** exhibited three quasi-reversible one-electron reduction waves due to a strong electronic communication between the tpy and the thienylene-ethynylene-based dendron (Figure 3a). Attachment of the tpy ligand shifted the oligothiophene oxidation wave of ligand **8** to a more positive value (Δ*E* ≈ 40 mV) compared to the terthiophene dendron [42]. The oxidation potential of the second-generation ligand **9** was also positively shifted by 30–60 mV compared to the corresponding dendron. This positive potential shift is the consequence of a stabilization of the tpy ligand. The broad and irreversible oxidation waves obtained for these ligands were assigned to multiple electron-transfer processes of the thienylene-ethynylene dendrons due to the presence of α-α and α-β conjugation pathways [42].

**Table 2:** Electrochemical properties of dendrons and dendrimers ^a^.

	*E*^0^_ox1_ [V]^a^	*E*^0^_ox2_ [V]	*E*^0^_red1_ [V]	*E*^0^_red2_ [V]	*E*^0^_red3_ [V]	HOMO [eV]^b^	LUMO [eV]^b^	*E*_g_^cv^ [eV]^c^

**8**^d^	0.84		−2.10	−2.35		−5.87	−3.11	2.76
**9**^d^	0.74	0.81	−1.84	−1.96	−2.30	−5.72	−3.33	2.39
**1**	0.85^d^	0.97^d^	−1.50^e^	−1.69^e^		−5.86	−3.68	2.18
**2**	0.81^d^	1.01^d^	−1.47^e^	−1.62^e^		−5.77	−3.72	2.05
[Ru(*tpy*)_2_][PF_6_]_2_^f^	0.92		−1.66	−1.89				

^a^Measured in CH_2_Cl_2_/TBAPF_6_ (0.1 M), *c* = 10^−3^ mol·L^−1^, 295 K, *V* = 100 mV·s^−1^ versus Fc/Fc^+^; ^b^set Fc/Fc^+^ *E*_HOMO_ = −5.1 eV; ^c^calculated from the onset potentials of the anodic and cathodic processes; ^d^measured in CH_2_Cl_2_ solution; ^e^measured in DMF as a solvent; ^f^taken from [48,52].

After complexation the oxidation potentials of the dendrons shifted to more positive values compared to the free ligand (Table 2). The Ru(III)/Ru(II) oxidation process occurred at 0.85 and 0.81 V for complexes **1** and **2**, respectively (Figure 3b). Typically, oxidation involves the removal of one electron from the ruthenium d orbital. A significant donor effect of the dendrons destabilizes the HOMO (dπ) orbitals of the metallodendrimers and consequently induces cathodic shifts on the quasi-reversible Ru(II) to Ru(III) oxidation waves compared to that of the parent [Ru(tpy)_2_]^2+^ complex (0.92 V versus Fc/Fc^+^) [52]. Beley et al. recently reported a similar cathodic shift of the oxidation potentials for thienyl- and bithienyl-substituted Ru-tpy complexes (*E*^0^_ox_ = 0.86 and 0.84 V versus Fc/Fc^+^, respectively) [48,53]. As described above, the irreversible oxidation processes for complexes **1** and **2** can be explained by the overlapping of multiple electron-transfer processes of the oligothienylene-ethynylene dendrons and Ru(III)/Ru(II) redox process. The cyclic voltammograms of both complexes **1** and **2** exhibited two reversible waves at negative potentials that correspond to the successive one electron redox processes of the two electroactive tpy groups. The reduction potentials of **1** and **2** were shifted to more positive values than that observed in parent [Ru(typ)_2_]^2+^. Therefore, for these complexes, the destabilization of the HOMO (d orbital of ruthenium) and the stabilization of the LUMO (π* orbital of the tpy-ligand) was observed. Close inspection of the data for the complexes (Table 2) revealed that the reduction of complex **1** requires more energy including a slightly larger peak-to-peak separation (Δ*E*_p_ = 190 mV) than that of second generation complex **2** (Δ*E*_p_ = 150 mV), which is ascribed to an increase of the localized tpy-thienylene-ethynylene → Ru donor character. The HOMO and LUMO energy levels of the ligands and complexes were estimated from the onset of oxidation and reduction potentials and are depicted in Table 2. With an increasing generation of the dendrons from G1 to G2 an increase in the HOMO energy level and decrease in the LUMO level was observed for both ligands and complexes. This trend was further reflected by the decrease in the band gap with increasing generation.

## Conclusion

The synthesis and characterization of shape-persistent oligothienylene-ethynylene dendrons of the first and second generation, which were core-functionalized with terpyridine (tpy) chelating ligands, and their corresponding Ru(II)-complexes are reported. The absorption, fluorescence and electrochemical studies of these metallodendrimers showed a very efficient energy delocalization over the extended π-system comprising the oligothienylene-ethynylene dendrons and the excited ruthenium-based component. The Ru(II)-complexes showed a very strong MLCT band with broad absorption covering the whole UV–vis region. The absorption spectra of the complexes feature a number of transitions, either localized on both the oligothienylene-ethynylene dendron and the tpy ligands, or of an MLCT (Ru→tpy) nature. These complexes exhibit a significantly red-shifted MLCT emission of Ru(II)-tpy complexes in comparison to the parent [Ru(tpy)_2_]^2+^ suggesting electronic energy transfer from oligothiophene dendrons to the Ru(II) center. The functionalization of these metal complexes with oligothienylene-ethynylene dendrons could open interesting perspectives for the study of the vectorial transport of energy or charges over very long distances. These metallodendrimers showed reversible redox characteristics typical of electroactive metal centers.

## Experimental

All starting materials were purchase from Aldrich, Merck and VWR chemicals. All solvents were dried before use. ^1^H and ^13^C NMR were measured in CDCl_3_ on a Bruker AMX 400 instrument at 400 and 100 MHz. Chemical shifts are denoted as δ in parts per million (ppm) and *J* values are given in hertz (Hz). GC-mass spectra were recorded with a Varian Saturn 2000 and MALDI–TOF mass spectra on a Bruker Daltonic Reflex III (dithranol as the matrix). Iodinated dendrons **6** and **7** were prepared according to literature [42].

**Photophysical measurements.** Optical measurements were carried out in one centimeter cuvettes with Merck Uvasol grade solvents. Absorption spectra were recorded on a Perkin Elmer Lambda 19 spectrometer and fluorescence emission spectra on a Perkin Elmer LS 55 spectrometer. Emission quantum yields of ligands and complexes were measured using 9,10-diphenylanthracene (Φ_f_ = 0.9 in cyclohexane) [54] and [Ru(bpy)_3_][PF_6_]_2_ (Φ_f_ = 0.062 in acetonitrile) [55] by using Equation 1,

[1]
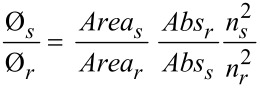


where *Abs* and *n* are the absorbance value at the excitation wavelength and the refractive index of the solvent, and *s* and *r* refer to sample and reference, respectively.

Cyclic voltammetry experiments were performed with a computer-controlled AutoLab PGSTAT30 in a three-electrode single compartment cell (2 mL). The platinum working electrode consisted of a platinum wire sealed in a soft glass tube with a surface area of *A* = 0.785 mm^2^, which was polished down to 0.5 μm with Buehler polishing paste prior to use in order to obtain reproducible surfaces. The counter electrode consisted of a platinum wire, and the reference electrode was an Ag/AgCl secondary electrode. All potentials were internally referenced to the ferrocene/ferricenium couple.

**Calculations.** Full geometry optimizations were performed in the gas phase by using density functional theory (DFT) calculations based on the B3LYP hybrid exchange-correlation functional together with the 6-31G^+^(d) basis sets [56]. Parallel optimizations were performed for the ligands by using the LANL2DZ basis set. The latter was used for the geometry optimization of the complexes. All calculations were performed by using the Gaussian 09 program package [57].

### Synthesis

4'-[5,5"-Bis(trimethylsilyl)-2,2':3',2"-terthien-5'-ylethynyl]2,2':6',2"-terpyridine (**8**). To a degassed suspension of Pd(PPh_3_)_2_Cl_2_ (2.73 mg, 3.89 μmol) and CuI (0.37 mg, 1.94 μmol) in diisopropylamine (10 mL) was added 4'-ethynyl-2,2':6',2"-terpyridine (**5**, 50 mg, 194 μmol) and iodinated G1-dendron **6** (110 mg, 213 μmol). The reaction mixture was stirred at 50 °C for 3–4 h and was then poured into water. The resulting aqueous solution was extracted with dichloromethane. The combined organic extracts were collected and dried over sodium sulfate. After removal of the solvent, the crude product was purified by column chromatography on basic aluminum oxide eluting with *n*-hexane/dichloromethane (2:1) to give 108 mg (0.17 mmol, 85%) of the desired product **8** as a yellow solid. Mp 170–172 °C; ^1^H NMR (CDCl_3_) 0.31 (s, 9H), 0.33 (s, 9H), 7.12 (d, *J* = 2.27 Hz, 1H), 7.13 (d, *J* = 2.53 Hz, 1H), 7.15 (d, *J* = 3.28 Hz, 1H), 7.19 (d, *J* = 3.54 Hz, 1H), 7.36 (ddd, *J* = 7.51, 4.73, 0.95 Hz, 2H), 7.38 (s, 1H), 7.87 (td, *J* = 7.70, 1.76 Hz, 2H), 8.56 (s, 2H), 8.61 (td, *J* = 7.83, 1.26 Hz, 2H), 8.73 (dd, *J* = 4.48, 0.94 Hz, 2H); ^13^C NMR (CDCl_3_) −0.13, −0.06, 86.58, 92.63, 121.20, 120.66, 122.38, 124.01, 128.16, 129.14, 131.83, 132.86, 134.16, 134.67, 135.73, 136.87, 139.20, 141.27, 141.58, 142.94, 149.20, 155.59, 155.64; MALDI–TOF *m*/*z*: [M^+^] calcd for C_35_H_33_N_3_S_3_Si_2_, 648.02; found, 648.23; Elemental analysis (%): Anal. calcd C, 64.87; H, 5.13; N, 6.48; S: 14.84; found: C, 64.73; H, 5.12; N, 6.41; S, 14.76.

4'-[5,5"-Bis{5,5"-bis(trimethylsilyl)-2,2':3',2"-terthien-5'-ylethynyl}-2,2':3',2"-terthien-5'-yl-ethynyl]2,2':6',2"-terpyridine (**9**). To a degassed suspension of Pd(PPh_3_)_2_Cl_2_ (2.73 mg, 3.89 μmol) and CuI (0.36 mg, 1.94 μmol) in diisopropylamine (10 mL) was added 4'-ethynyl-2,2':6',2"-terpyridine (**5**, 50 mg, 194 μmol) and iodinated G2-dendron **7** (257 mg, 21 mmol). The reaction mixture was stirred at 50 °C for 3–4 h and was then poured into water. The resulting aqueous solution was extracted with dichloromethane. The combined organic extracts were collected and dried over sodium sulfate. After removal of the solvent, the crude product was purified by column chromatography on aluminum oxide eluting with *n*-hexane/dichloromethane (3:2) and subsequently with dichloromethane/methanol (96:4) to give 243 mg (0.18 mmol, 94%) of the desired product **9** as a yellow solid. Mp 92–94 °C; ^1^H NMR (CDCl_3_, δ ppm) 0.30 (s, 18H), 0.31 (s, 18H), 7.04 (d, *J* = 3.79 Hz, 1H), 7.08–7.14 (m, 7H), 7.17 (d, *J* = 3.28 Hz, 2H), 7.20 (d, *J* = 3.78 Hz, 1H), 7.24 (d, *J* = 3.79 Hz, 1H), 7.32, (d, *J* = 3.53 Hz, 2H), 7.35 (s, 1H), 7.37 (dd, *J* = 7.2, 5.18 Hz, 2H), 7.88 (td, *J* = 7.70, 1.68 Hz, 2H), 8.57 (s, 2H), 8.63 (d, *J* = 8.08 Hz, 2H), 8.73 (d, *J* = 4.04 Hz, 2H); ^13^C NMR (CDCl_3_, δ ppm) −0.139, −0.071, 85.96, 86.99, 87.28, 87.46, 87.74, 93.26, 120.84, 121.02, 121.23, 121.69, 122.40, 123.59, 124.11, 124.59, 127.63, 128.02, 128.03, 128.24, 128.96, 128.98, 131.36, 131.63, 131.65, 132.51, 132.55, 132.64, 133.73, 133.90, 134.13, 134.15, 134.33, 134.87, 134.98, 135.42, 135.57, 136.99, 137.92, 139.24, 139.29, 141.06, 141.10, 141.61, 141.66, 142.67, 142.72, 149.16, 155.42, 155.53; MALDI–TOF *m*/*z*: [M^+^] calcd for C_69_H_61_N_3_S_9_Si_4_, 1333.20; found, 1333.50; Elemental analysis (%): Anal. calcd C, 62.16; H, 4.61; N, 3.15; S, 21.65; found: C, 62.02; H, 4.58; N, 3.08; S, 21.69.

Bis[4'-{5,5"-bis(trimethylsilyl)-2,2':3',2"-terthien-5'-ylethynyl}2,2':6',2"-terpyridine-κN1,κN1',κN1"]ruthenium(II) hexafluorophosphate (**1**). To a solution of ligand **8** (100 mg, 154 μmol) in THF/MeOH (1:2, 15 mL) was added [Ru(DMSO)_4_(Cl)_2_] (37.3 mg, 77.1 μmol), and then the suspension was heated at 60 °C for 36 h. After completion of the reaction the mixture was cooled, and the resulting deep red solid was filtered and washed with cold methanol and then with diethyl ether. The crude product was then purified by column chromatography (SiO_2_; acetonitrile/H_2_O/KNO_3_ (aqueous sat.) 7:0.5:0.5). The resulting complex was further dissolved in dichloromethane and precipitated by the addition of a methanolic solution of ammonium hexafluorophosphate to afford the desired complex **1** as a red solid (156 mg, 92.5 μmol; 60%). Mp > 300 °C; ^1^H NMR (CD_2_Cl_2_) 0.34 (s, 18H), 0.35 (s, 18H), 7.18–7.30 (m, 12H), 7.37 (td, *J* = 0.50, 5.43 Hz, 4H), 7.66 (s, 2H), 7.94 (td, *J* = 7.89, 1.35 Hz, 4H), 8.42 (d, *J* = 8.33 Hz, 4H), 8.70 (s, 4H); ^13^C NMR (CD_2_Cl_2_) 0.10, 0.19, 91.83, 92.31, 119.74, 125.14, 125.20, 128.88, 129.22, 130.19, 131.61, 132.96, 134.93, 134.97, 137.19, 138.15, 139.01, 139.22, 141.56, 142.51, 144.43, 152.75, 155.45, 157.72; MALDI–TOF *m*/*z*: [M^+^] calcd for C_70_H_66_N_6_RuS_6_Si_4_·2PF_6_, 1687.11; found, 1397.2 [M^+^ − 2PF_6_^−^], 1541.6 [M^+^ − PF_6_^−^]; Elemental analysis (%): Anal. calcd for C, 49.84; H, 3.94; N, 4.98; found: C, 49.92; H, 3.90; N, 5.05.

Bis[4'-{5,5"-bis(5,5"-bis(trimethylsilyl)-2,2':3',2"-terthiophen-5'-ylethynyl)-2,2':3',2"-terthiophen-5'-ylethynyl}2,2':6',2"-terpyridine-κN1,κN1',κN1"]ruthenium(II) hexafluorophosphate (**2**). To a solution of G2-ligand **9** (100 mg, 75 μmol) in THF/MeOH (1:2, 15 mL) was added [Ru(DMSO)_4_(Cl)_2_] (18.1 mg, 37.5 μmol), and then the suspension was heated at 60 °C for 48 h. After completion of the reaction the mixture was cooled, and the resulting deep red solid was filtered and washed with cold methanol and then with diethyl ether. The crude product was then purified by column chromatography on aluminum oxide by gradient elution (dichloromethane → dichloromethane/MeOH (95:5)). The complex was further dissolved in dichloromethane and precipitated by the addition of a methanolic solution of ammonium hexafluorophosphate to afford the desired complex **2** as a red solid (126 mg, 41.2 μmol; 55%). Mp > 300 °C; ^1^H NMR (CD_2_Cl_2_) 0.30 (s, 36H), 0.31 (s, 36H), 7.12 (d, *J* = 3.03 Hz, 4H) 7.13–7.17 (m, 10H), 7.18 (dd, *J* = 3.53, 0.50 Hz, 4H), 7.21 (d, *J* = 4.04 Hz, 2H), 7.28–7.29 (m, 7H), 7.31 (d, *J* = 3.79 Hz, 2H), 7.33 (d, *J* = 5.30 Hz, 3H), 7.38 (dd, *J* = 5.43, 0.63 Hz, 4H), 7.66 (s, 2H), 7.94 (dt, *J* = 7.83, 1.34 Hz, 4H), 8.44 (d, *J* = 8.32 Hz, 4H), 8.72 (s, 4H); ^13^C NMR (CD_2_Cl_2_) 0.10, 0.17, 87.58, 87.66, 87.90, 88.48, 91.70, 92.37, 120.95, 121.29, 121.49, 124.36, 125.25, 125.61, 128.77, 128.85, 128.87, 128.90, 129.51, 129.83, 129.85, 131.43, 132.49, 132.52, 132.53, 133.36, 133.45, 134.53, 134.77, 134.83, 134.86, 135.54, 135.72, 135.86, 136.66, 137.88, 138.26, 139.04, 139.54, 139.58, 141.97, 142.02, 142.05, 142.08, 143.76, 143.81, 152.76, 152.78, 155.48, 157.72; MALDI–TOF *m*/*z*: [M^+^] calcd for C_138_H_122_N_6_RuS_18_Si_8_·2PF_6_, 3057.43; found, 2767.5 [M^+^ − 2PF_6_^−^], 2908.4 [M^+^ − PF_6_^−^]; Elemental analysis (%): Anal. calcd for C, 54.21; H, 4.02; N, 2.75; found: C, 54.37; H, 3.86; N, 2.72.
